# Fabrication, Characterization and Performance of Low Power Gas Sensors Based on (Ga_x_In_1-x_)_2_O_3_ Nanowires

**DOI:** 10.3390/s21103342

**Published:** 2021-05-11

**Authors:** Elena López-Aymerich, Guillem Domènech-Gil, Mauricio Moreno, Paolo Pellegrino, Albert Romano-Rodriguez

**Affiliations:** 1 Institut de Nanociència i Nanotecnologia (IN2UB), Universitat de Barcelona, Carrer Martí i Franquès 1, E-08028 Barcelona, Spain; elenalopez@ub.edu (E.L.-A.); guillemdom@gmail.com (G.D.-G.); mauricio.moreno@ub.edu (M.M.); 2 Departament d’Enginyeria Electrònica i Biomedical, Universitat de Barcelona, Carrer Martí i Franquès 1, E-08028 Barcelona, Spain; 3 Department of Physics, Chemistry and Biology, IFM, Linköping University (LiU), 581 83 Linköping, Sweden

**Keywords:** gas sensor, metal oxide, nanowires

## Abstract

Active research in nanostructured materials aims to explore new paths for improving electronic device characteristics. In the field of gas sensors, those based on metal oxide single nanowires exhibit excellent sensitivity and can operate at extremely low power consumption, making them a highly promising candidate for a novel generation of portable devices. The mix of two different metal oxides on the same nanowire can further broaden the response of this kind of gas sensor, thus widening the range of detectable gases, without compromising the properties related to the active region miniaturization. In this paper, a first study on the synthesis, characterization and gas sensing performance of (Ga_x_In_1-x_)_2_O_3_ nanowires (NWs) is reported. Carbothermal metal-assisted chemical vapor deposition was carried out with different mixtures of Ga_2_O_3_, In_2_O_3_ and graphite powders. Structural characterization of the NWs revealed that they have a crystalline structure close to that of In_2_O_3_ nanowires, with a small amount of Ga incorporation, which highly depends on the mass ratio between the two precursors. Dedicated gas nanosensors based on single NWs were fabricated and tested for both ethanol and nitrogen dioxide, demonstrating an improved performance compared to similar devices based on pure In_2_O_3_ or Ga_2_O_3_ NWs.

## 1. Introduction

In the last four decades, gas sensors have undergone extraordinary development, driven by their paramount role in indoor/outdoor environmental and automotive/industrial air monitoring. Now, numerous sensing devices are commercially available. However, significant potential remains for further performance improvements; thus, a large research effort is currently underway aimed at additional development. Among the different classes of available devices, those relying on semiconducting metal oxides (MOx) as the sensing material are particularly popular [1], due to their low cost, robustness and high sensitivity. Their sensing principle is based on the variation of the electrical resistance when in the presence of certain gases [2,3,4]. The driver of this sizeable change is the interaction of the O-atoms at the semiconductor’s surface with the adsorbed gas molecules. Nevertheless, in general, this class of devices presents some drawbacks, such as poor selectivity towards a gas mixture and the requirement of a high operating temperature, with the consequent large power consumption.

Currently, one of the active research areas in gas sensor development is aimed at the miniaturization of the MOx sensing region of the device [5,6,7]. This can be achieved by reducing the device’s dimensions, with the consequent increase in the surface-to-volume ratio, which results in a larger response of the sensing material and a reduction in the device’s power consumption.

With this aim, the potential performance of MOx nanowires (NWs) acting as the sensing element of these devices has been observed. Among different MOx materials, In_2_O_3_ [4] and Ga_2_O_3_ [8] have been demonstrated to be efficient gas sensing materials that can be grown in NW form via chemical vapor deposition (CVD) routes [9]. The alloy of these two semiconductors in thin film form has been reported in the literature [10,11], whereas their growth in a NW geometry has been scarcely studied [12,13]. To the best of our knowledge, only one short report on the gas sensing properties of this material has been made available; this report was published by our group [14]. These preliminary findings motivated us to explore in depth the feasibility of fabricating this material in the form of nanowires and validating its use as the active component in low-power gas sensing devices.

The present work is focused on the fabrication of gas sensors based on (Ga_x_In_1-x_)_2_O_3_ NWs. The active material was grown by means of the Vapor–Liquid–Solid (VLS) mechanism. Several Ga-to-In precursor ratios were studied, in addition to other synthesis conditions. Morphological, structural and optical characterizations were carried out using scanning electron microscopy (SEM), X-ray diffraction (XRD), transmission electron microscopy (TEM) and photoluminescence (PL). Finally, an initial study is presented of the sensing response for ethanol and nitrogen dioxide, and the results are compared with those obtained by In_2_O_3_ and Ga_2_O_3_ NWs, thus highlighting the advantages of using this compound as the active material in gas sensors.

## 2. Materials and Methods

### 2.1. Chemical Vapor Deposition Growth

One of the most widely used techniques for the growth of NWs and other types of one-dimensional nanostructures is based on the so-called Vapor–Liquid–Solid (VLS) crystal growth mechanism, first described for the growth of silicon by Wagner and Ellis in 1964 [15,16]. This mechanism relies on the formation of a liquid eutectic alloy droplet via the incorporation of a precursor material from a gas phase into a liquid metal catalyst at high temperature that, once achieving a supersaturation regime, acts as a NW nucleation site. This nucleation is ruled by the precipitation of the precursor material at the liquid–solid interface, giving rise to the NW crystallization, for which growth direction and diameter are controlled mainly by the dimensions of the catalyst droplet, among other parameters.

**Figure 1:**
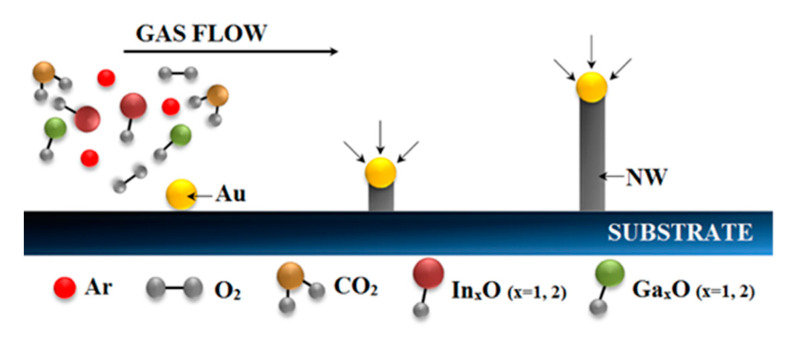
Sketch of the VLS growth process showing the nucleation and the progressive growth of the NWs via the incorporation of the materials in the catalytic gold droplet.

This growth process takes place in two steps: a fast initial, catalyst-driven, growth in length and a subsequent process, which is slower due to the absence of the catalyst on the walls of the NW, in the axial direction [15]. Both regimes are maintained until the extinction of the precursor in the gas phase or the lowering of the sample’s temperature occurs. This sequence is briefly summarized in Figure 1.

VLS growth was carried out in a quartz tube with a diameter of 5 cm inserted into a Lindbergh furnace with three-independent-zone temperature control, and connected to a gas injection system at one end and a primary pump at the other. The precursor material used was a mixture of Ga_2_O_3_ (purity 99.999%) and In_2_O_3_ (purity 99.9%) micro-powders and graphite (Ga_2_O_3_: In_2_O_3_: C). The materials were mixed for 15 min in an agate mortar. For each experiment, 0.3 g of the precursor was placed inside an alumina boat close to the left end of the quartz tube. A constant flux of carrier gas, argon in our case, transported the evaporated precursor along the tube where Au (catalyst) sputter-covered Si/SiO_2_ substrates (5 × 5 mm^2^ pieces cut from a thermally oxidized Si wafer with 0.5–0.7 μm-thick SiO_2_) were uniformly distributed on top of four alumina plates.

**Figure 2:**
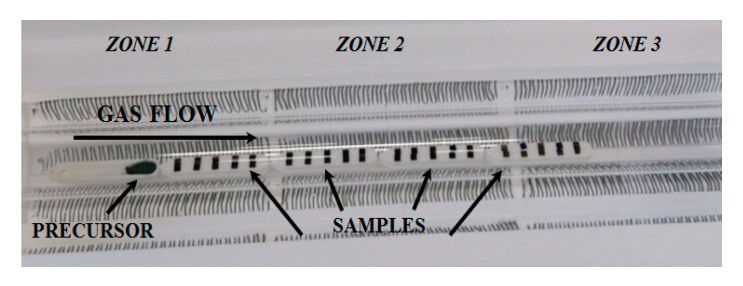
Image of the precursor and sample distribution inside the quartz tube where the synthesis experiments were carried out.

The Au film was sputter-covered for 20 s, creating a discontinuous layer with a thickness of a few nanometers. The first step of the NW synthesis was the evaporation of the precursor mixture and, for this, the temperature was increased to 950 °C in the first zone of the tube. Due to the high temperature of the chamber, the Au was found in liquid phase on top of the Si/SiO_2_ substrates, forming nanometric spherical droplets due to a dewetting process [17]. The thickness of this layer strongly determines the size and the density of the gold droplets. The exact layout of the experiment, including precursor and sample distribution, is shown in Figure 2, where the carrier gas, injected from the left side, flows downstream.

Different mass ratios of Ga_2_O_3_: In_2_O_3_: C precursors were used in the present study to span different Ga-to-In NW compositions; for example, 2:1:6, 1:6:12 and 10:1:20. Considering the molecular weight of each precursor material, these weight proportions correspond to an Ga-to-In atomic ratio of approximatively 1:1 (Sample A), 1:9 (Sample B) and 6:1 (Sample C), while maintaining a constant metal-oxide-to-carbon ratio. The addition of graphite enables the evaporation temperature to be decreased via a carbothermal reduction in both In_2_O_3_ and Ga_2_O_3_. By the carbo–thermal reaction, the metal oxides are reduced by the oxidation of carbon, which forms either CO or CO_2_.

First, the chamber was vacuum pumped down to a few Pa to remove the oxygen inside the tube. After this step, which typically lasted 10 min, the pumping was stopped and the gas flow injection system, composed of four MKS Mass-Flow controllers, set a constant flow of 100 sccm (standard cubic centimeters) Ar (purity 5N) or Ar / O_2_ gas mixture to transport the precursor in gas phase towards the substrates. Consequently, the pressure in the tube increased to atmospheric level and was kept at this value for the rest of the process. Different experiments were carried out by changing both the O_2_ amount from 0 to 10% of the overall gas concentration, and the duration of the process, between 1 and 2 h, to obtain a set of optimized synthesis parameters. The chamber temperature was controlled at the three different zones of the tube, with the precursor boat maintained at 950 °C, and the substrates were maintained at a different temperature between 850 and 950 °C. Cooling was allowed to proceed naturally, typically taking about 3 h.

Additional experiments (Sample D) were carried out using 0.25 g of a precursor based on a mix of powders of Ga_2_O_3_ and graphite, with a composition of 1:1.5, and using a quartz tube in which In_2_O_3_ NWs were previously grown. In this way, the remaining In_2_O_3_ on its walls was used to provide the indium to the compound. Using these additional runs, we aimed to evaluate any possible effect on the synthesis caused by the progressive accumulation of by-products deposited on the sidewalls of the chamber, and to estimate the Ga content in the samples under these extreme conditions, even if in an uncontrolled manner. Two experiments were carried out under these conditions using pure Ar as a carrier gas, one for 2 h and another for 5 h, with the chamber operating at 950 °C at the precursor’s zone, and between 800 and 850 °C at the samples’ zone.

### 2.2. Active Material Characterisation

All the substrates used in the deposition of the material were first inspected by means of a Jeol 7001 or a Jeol 7100 Field Emission Scanning Electron Microscopes (FE-SEM) (Jeol Ldt., Osaka, Japan), both operating at 15 kV. This allowed the morphology of each sample to be studied and correlated to the specific growing conditions employed in the run, leading to the determination of the optimal conditions for the growth of the NWs.

Selected samples were further investigated by X-ray diffraction (XRD), photoluminescence (PL) spectroscopy, and transmission electron microscopy (TEM) analysis. XRD analysis was performed using a PANalytical X’Pert PRO MRD diffractometer (Malvern Panalytical Ltd., Malvern, UK), operating with K_α_ radiation of a Cu anode, with a wavelength of 0.154184 nm. To maximize the interaction of the X-ray beam and the grown NW assembly, a grazing incidence (GIXRD) configuration was chosen, with the X-ray beam incident at an angle of 0.5° to the surface. PL measurements were carried out in backscattering configuration, by exciting the samples with a 325 nm He-Cd laser, providing an optical power of about 16 mW, as measured on the sample’s surface. The PL signal was determined in the 330–900 nm optical range by an Acton SP2750 double grating monochromator (Princeton Instruments, Acton, MA, USA) and a photomultiplier (Hamamatsu, Shizuoka, Japan). The TEM analysis was carried out using a Jeol J2100 transmission electron microscope (Jeol Ltd., Osaka, Japan), operating at 200 kV.

### 2.3. Gas Measurements

To perform the gas sensor measurements, a single NW was placed in contact with the top of a micro-hotplate (MHP). To achieve this configuration, the grown material was first removed from its substrate by sonicating it in a cyclohexane (C_6_H_14_) solution, resulting in a suspension containing NWs. Two droplets of 0.5 µL of this solution were deposited onto the MHP, leaving several NWs on it after evaporation. The MHP device consisted of a suspended thin membrane, connected to the silicon substrate by 4 arms, fabricated using bulk micromachining processes. A serpentine-shaped buried Pt circuit acted as a resistive heater, allowing an operating temperature up to 350 °C to be reached, while maintaining a power consumption of no more than 6 mW. A suitable SiO_2_ protecting layer separated the buried heater from the interdigitated Ti/Pt electrodes, which were defined on top of the MHP, where the NWs were deposited [18], using photolithography in combination with lift-off. Single NWs, located in the proximity of the electrodes, acted as the quantitative transducer in the presence of detectable gases. For this purpose, one of the NWs was placed in contact by platinum deposition using a FEI Helios Nanolab 650 (FIB) dual-beam instrument, using a trimethylcyclopentadienyl-platinum ((CH_3_)_3_CH_3_C_5_H_4_Pt) precursor, which was decomposed by the primary electron source. A detailed explanation of the contact fabrication procedure can be found elsewhere [19].

To perform the sensor tests, the MHP was first mounted, glued, and ball bonded to a TO-8 holder. Then, electrical characterization tests were carried out by allowing current to flow both through the contacted NW and through the heater, independently. The latter allowed the MHP temperature to be controlled. During the gas measurements, the NW current was maintained at 5 nA to prevent overheating and damage of the NW.

Finally, gas sensing measurements were carried out inside a stainless-steel gas chamber with a volume of 8.6 ml and connected to a gas mixer based on 4 Bronkhorst Mass Flow Controllers, allowing the mixture of Synthetic Air (SA, 79 % N_2_ and 21 % O_2_) and other gases, ethanol and NO_2_. These gases were diluted in SA in suitable concentrations up to 100 ppm. The NW resistance and MHP heating power were recorded by means of a Keithley 2602A dual source measurement unit. The whole process of gas introduction in the chamber and the electrical measurements was controlled by dedicated home-developed LabVIEW software.

## 3. Results and Discussion

### 3.1. Scanning Electron Microscopy (SEM)

**Figure 3:**
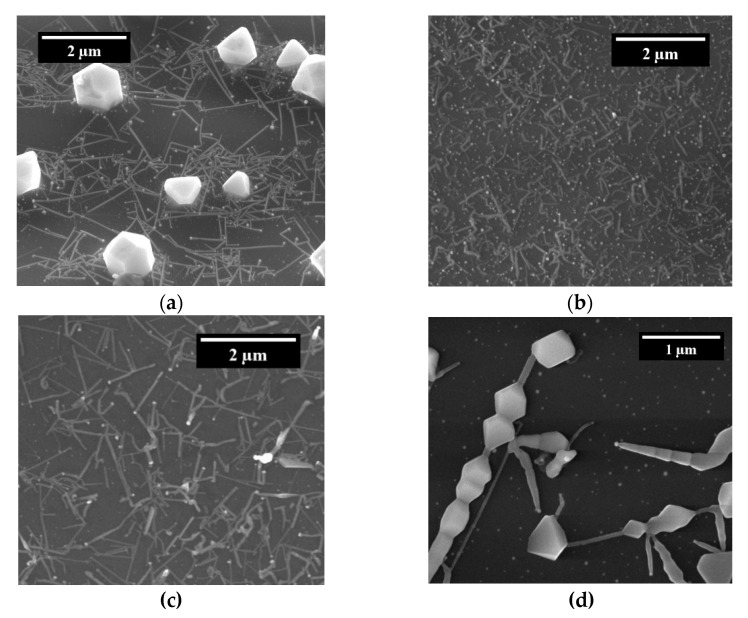
SEM images taken from samples A (**a**), B (**b**), C (**c**), and D (**d**), showing the resulting NW synthesis. The lighter spot at one end of the NWs indicates the gold droplet which acted as a catalyst. In addition, as a function of the oxygen concentration in the precursor and the temperature of the sample, sometimes either octahedra or nanorods can also be observed, as shown in (**a**) and (**d**).

By means of SEM imaging, the fabrication of NWs was revealed, with a length between 1 and 3 μm and a diameter around 100 nm, irrespective of the chosen precursor mixture. Figure 3 shows representative SEM images corresponding to the four experimental conditions explored in the present study. The presence of the gold droplet at the tip of the NWs confirms that the latter were formed according to the VLS mechanism previously described. In some images, it can be observed that not all NWs display a straight shape, but some of them grow in a zig-zag manner.

This is a result of the random diffusion of the highly mobile Au droplets over the substrate during the NW synthesis due to the high temperature attained in the process. In addition, some other NWs show a kink at the interface between the NW and the gold droplet at the tip. This finding implies that the formation of an island of material at the interface with the gold drop was favored during the thermal treatment, instead of an ordered, layer-by-layer growth [20]. Due to this change in the growth conditions, the NWs tend to bend or kink.

The coexistence of NWs with other nanostructures, such as nanorods (NR) or micro- or nanooctahedra, was also observed in some cases. The latter two can be described as a product of a Vapor–Solid (VS) growing mechanism that can be related to the high concentration of In in the environment [17], a process that competes with the catalyst-mediated VLS growth. Alternatively, the appearance of NRs, such as those shown in Figure 3d, indicates the simultaneous combination of VLS and VS mechanisms, in which a NW containing octahedra along the growth direction can be formed [21]. Regardless, both the morphology and dimensions of the obtained NWs, and the presence of more complex nanostructures, strongly resemble the typical scenario found in similar runs in which only In_2_O_3_ powers were used, pointing to a predominant presence of this material in the fabricated structures [4].

### 3.2. Transmission Electron Microscopy (TEM)

**Figure 4:**
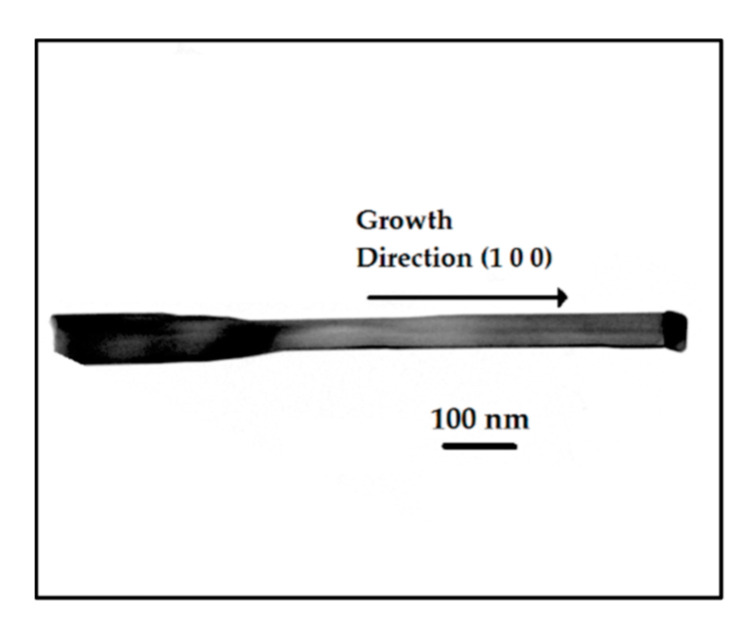
TEM image of a NW from Sample A, with an indication of its growth direction.

**Figure 5:**
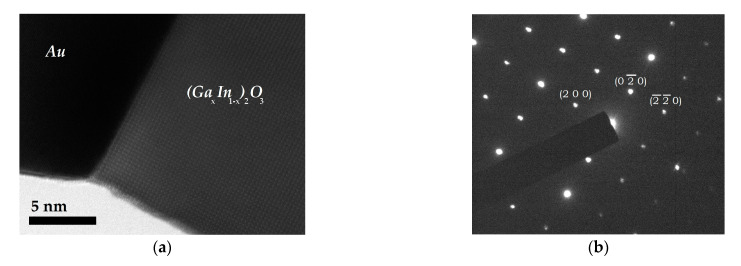
(**a**) High resolution TEM image of the interface between NW of the Sample A and its gold tip and (**b**) the corresponding diffraction pattern of the NW, with some of its peaks indexed. The diffraction pattern is coherent with cubic In_2_O_3_, observed along its zone axis [001].

TEM analysis was carried out for a selected subset of samples. A typical result of the observation is that shown Figure 4 and Figure 5, for a single nanowire from Sample A. The bright field image in Figure 4 reveals a straight NW with a typical length of about 1 µm. At the top right end, the darker Au tip can be clearly distinguished from the brighter nanowire structure, in agreement with the structures observed in SEM. Moreover, a variation is apparent in the NW diameter along its length. This occurrence may imply a variation in the experimental conditions (temperature, gas flow, gas partial pressure) during the NW growth and a limited diffusion length of adsorbed MOx species onto the surface, and thus tapering [22]. Finally, in the high-resolution image shown in Figure 5a, the crystallographic planes of the NW can be clearly observed, suggesting a long-range crystalline order of the fabricated structure.

Figure 5b shows a well-defined electron diffraction pattern from the main body of the NW, which confirms that the NW is monocrystalline. In addition, the indexation of the pattern was carried out under the reasonable hypothesis of a cubic phase, allowing full identification of the crystallographic planes and the zone axis, which is [001]. By comparing the NW images and the diffraction patterns from several analyzed NW, the preferential growth direction was determined to be <1 0 0>, in agreement with what reported for In_2_O_3_ NW growth [4]. This can also be identified in Figure 5, in which the growth direction, perpendicular to the eutectic/NW interface, is parallel to the (200) reciprocal lattice direction.

### 3.3. Photoluminescence (PL)

**Figure 6:**
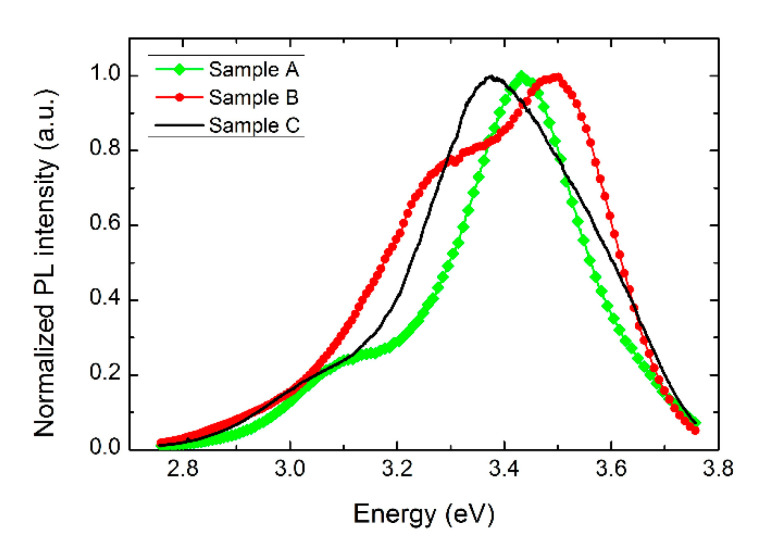
Normalized PL intensity spectrum from the three synthesized samples with controlled composition.

Photoluminescence characterization provides useful information about the optical bandgap of the material and about the density of optically active defects, which usually introduce discrete levels or bands inside the electronic bandgap of the semiconductor material. Typical PL responses of three different samples containing NWs are depicted in Figure 6. Here the spectra were normalized to allow a better comparison. The PL intensity from each sample is comparable, an additional indication that the density of the synthesized material is similar from sample to sample. Of the whole measured spectrum, we focused our attention on the relevant region, where the emission from the NWs reveals sizeable structures.

In the energy range from 2.8 to 3.8 eV, a broad band appears for all of the measured samples, with a line shape characteristic of each sample. To analyze each spectrum, we applied a fitting procedure using Gaussian bands, which were able to reproduce the measured spectra. In this way it is possible to discover the presence of two overlapping bands, one placed in the UV range at about 3.5–3.4 eV, the other one at lower energies, around 3.3–3.1 eV. As a function of the increasing Ga_2_O_3_ content in the precursor, it is possible to observe a progressive shift of the whole structure towards lower energies. This occurrence is followed by a weakening of the low energy band, which becomes broader and less intense. An additional finding is that the spectrum of Sample B closely matches that measured (not shown) from an In_2_O_3_ NW assembly excited under the same conditions.

To understand the observed behavior, a comparison was required with the available PL data for In_2_O_3_ NWs. For this kind of material, a general agreement was found regarding the assignment of the high energy band to the direct band-to-band transition, with an energy value of the optical bandgap slightly lower than that in bulk material; for example 3.5 eV vs. 3.7–3.8 eV [23]. Still under debate is the assignment of the low energy band, which some authors believe is related to electronic transitions involving the large density of oxygen vacancies generally found in this metal oxide [24], whereas others claim it corresponds to surface states [25]. Regardless, the observed energy shift of the PL bands with increasing Ga content would imply a band-gap narrowing, as already observed in bulk material [26], in addition to a suppression of defects, either O-vacancies or surface states, by the progressive incorporation of Ga into the material.

### 3.4. X-ray Diffraction (XRD)

**Figure 7:**
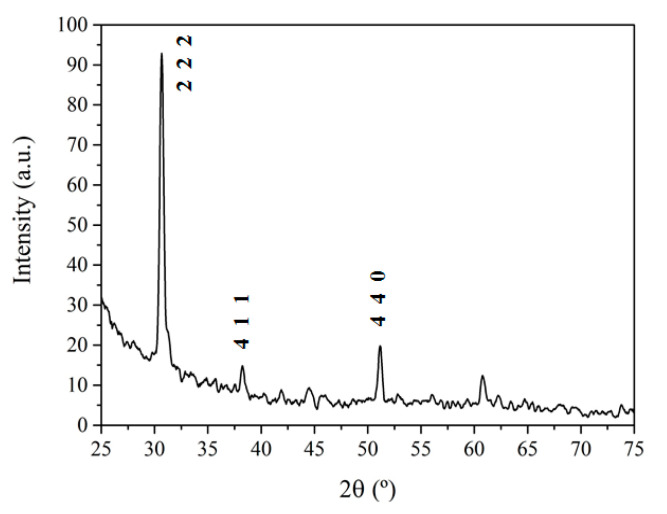
Measured XRD spectrum of (Ga_x_In_1-x_)_2_O_3_ NWs from Sample A. The peaks are labelled assuming a cubic phase close to the In_2_O_3_ known spectrum.

Figure 7 shows a typical XRD spectrum from this study, in this case obtained from Sample A.

**Figure 8:**
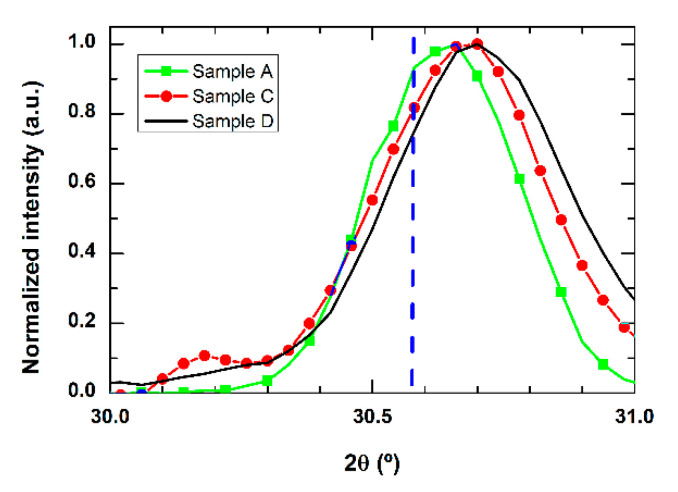
Magnification of the (2 2 2) peak for different XRD spectra of the evaluated samples. The line indicates the position of the In_2_O_3_ NWs corresponding peak.

The overall fingerprint of the observed structures closely matches that reported for cubic In_2_O_3_, whereas none of the typical features assigned to any known phase of Ga_2_O_3_ can be observed, within the available resolution. However, with respect to the XRD spectrum from the reference In_2_O_3_ NWs, a small but sizeable shift and a broadening of the diffraction peaks can be observed, as shown in Figure 8 for the (2 2 2) diffraction peak.

This is true for all of the samples, except from sample B, which is the one fabricated starting from the largest amount of indium in the precursor mixture, and for which the possible shift and broadening are well within the experimental error of the measurement. The magnitude of the deviation from the signature of pure In_2_O_3_ NWs is highly dependent on the composition of the mixture used as a precursor during the growth; that is, on the relative presence of Ga_2_O_3_ in the starting material, and, thus, the corresponding presence of Ga in the final product.

All of the different characterization techniques we used were consistent in establishing that the grown material has structural and compositional properties close to those of In_2_O_3_ NWs. At this stage, we attempted a quantitative estimation of the Ga content in the fabricated NWs, which allows the observed shift in XRD spectra to be justified as a function of the starting composition. The main hypothesis ruling this estimation is that the resulting material is mainly an In_2_O_3_ crystalline cubic lattice, in which a small quantity of Ga ions was incorporated in substitutional In sites. This results in a slightly distorted, more compressed lattice, because the ionic radius of Ga is smaller than that of In [27], accounting for the XRD shift towards larger diffraction angles. For each measured sample, using Bragg’s law, the XRD angle corresponding to the (2 2 2) diffraction peak can be converted into the corresponding lattice parameter *a*. At this point we followed an interpolation scheme, similar to Vegard’s law. The interpolation of the reference data for cubic In_2_O_3_ and Ga_2_O_3_ strictly cannot be applied in this case, because the two metal oxides belong to different crystalline space groups. Thus, the measured lattice parameter *a* was a linear function of the fraction *x* of Ga into the compositional formula (Ga_x_In_1-x_)_2_O_3_ according to:

(1) $$a=a_{Ga_{2}O_{3}}\cdot x+a_{In_{2}O_{3}}\cdot \left(1-x\right),$$

where $a_{In_{2}O_{3}}=1.0118\;\mathrm{nm}$ is the reported value for the In_2_O_3_ lattice parameter [28], and $a_{Ga_{2}O_{3}}=0.9779\;\mathrm{nm}$ is the calculated value when replacing each In ion with Ga in the indium oxide lattice. The whole set of evaluated parameters is summarized in Table 1.

According to this estimation, the results show that the percentage of Ga as a metallic ion in the compound ranges from less than 0.1% to 9%, when increasing Ga_2_O_3_ in the mixture. These values are much lower than the Ga relative amount in the precursors. This behavior can be understood by looking at the difference in formation enthalpy for both oxides (ΔH^°^In_2_O_3_ = −222.47 kcal/mol and ΔH^°^Ga_2_O_3_ = −259.90 kcal/mol [29]), which results in a much larger In_2_O_3_ reduction, and, in turn, a larger evaporation rate and incorporation into the Au catalyst for In ions with respect to Ga ions. This remained true even in the case of Sample C, where a much larger Ga_2_O_3_ concentration exists in the precursor. A similar explanation can be used to describe the results for Sample D, for which it was not possible to control the Ga-to-In ratio. In this case, the introduction of substitutional Ga ions reaches its highest value because, with respect to the other samples, In_2_O_3_ evaporation is less effective because it is not carbo-thermally assisted, and the relative amount of Ga_2_O_3_ is also much larger.

One has to be aware of the approximations made in all of these calculations, which lead to a semi-quantitative estimation. For instance, we disregarded any possible lack of uniformity in the preferential location of Ga ions in the matrix, in addition to any variation in defect density related to the Ga incorporation. Thus, the Ga relative concentrations we estimated have to be considered upper limits, indicating that Ga content in the NW is around a few percent. Nonetheless, these results are consistent with all of our experimental observations and close to those found in other studies, in which similar Ga concentrations were attained in Ga-enriched indium oxide [26,30].

### 3.5. Gas Sensing Behaviour

**Figure 9:**
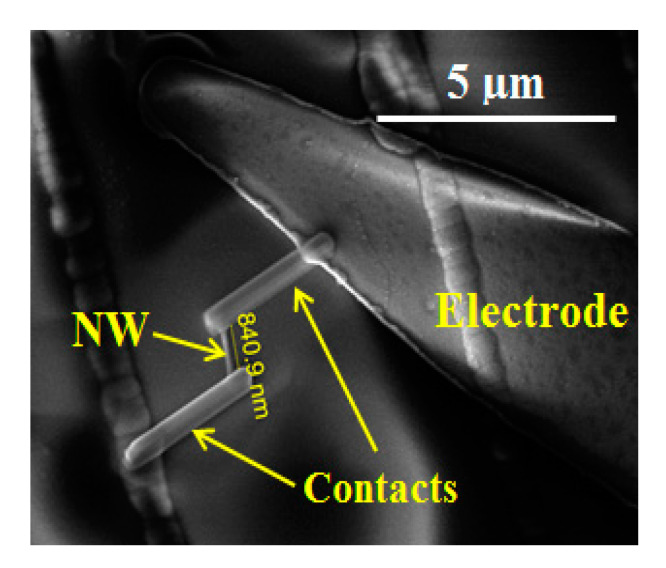
SEM image of the active region of the sensor, where a single NW is placed in contact with the electrode arms deposited onto the upper surface of the micro-hotplate.

**Figure 10:**
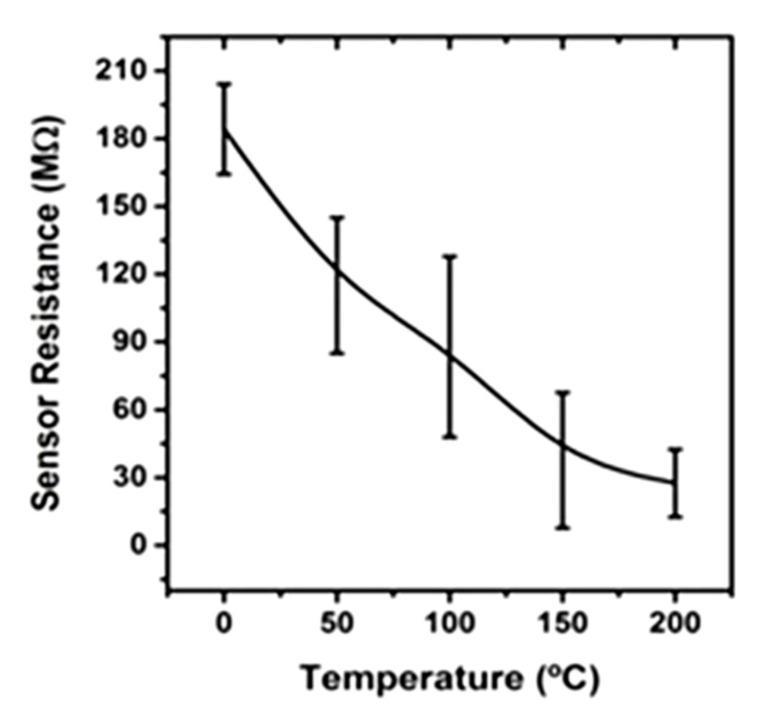
Sensor resistance of a single NW from Sample A (at a fixed current of 5 nA) as a function of the heater temperature.

The synthesized NWs were the active material in test structures we fabricated to evaluate them as low-power gas sensors. Figure 9 shows an image of the active region of the sensor, in which one single NW was placed in contact with the electrodes. Prior to the gas sensing measurement, the calibration resistance-to-temperature curve was measured for one of the bonded NWs. The results were obtained by varying the heater temperature while maintaining a 5 nA constant current flowing through the NW, as shown in Figure 10. Because the sensor resistance decreased as the temperature rose, the semiconductor behavior of the NW was confirmed. Because both In_2_O_3_ and Ga_2_O_3_ are generally n-type semiconductors, it is expected that the synthesised (Ga_x_In_1-x_)_2_O_3_ NWs would also have the same characteristic.

Furthermore, during the gas sensor operation, modification of the resistance occurs when the gas is introduced into the measuring chamber. Accordingly, because the material is an n-type semiconductor, in the presence of a reducing gas, such as ethanol, the NW resistance decreases, whereas the opposite behavior is observed in the presence of an oxidizing gas, such as NO_2_.

The two main parameters which account for the quality of the sensing material are the sensor response and the response time. The sensors’ response can be expressed in this work as:

(2) $$S_{\left(\%\right)}=\frac{\left|R_{SA}-R_{gas}\right|}{R_{SA}}\cdot 100,$$

where $R_{SA}$ is the resistance measured in the SA environment, and $R_{gas}$ is the resistance measured in the gas–SA mixture environment. The response time τ_res_ is defined as the time span between 10% and 90% of the baseline-steady state resistance variation, whereas the recovery time τ_rec_ is the lapse needed to pass from 90% to 10% of the steady state baseline-change during the transient to recover the operating resistance under SA.

**Figure 11:**
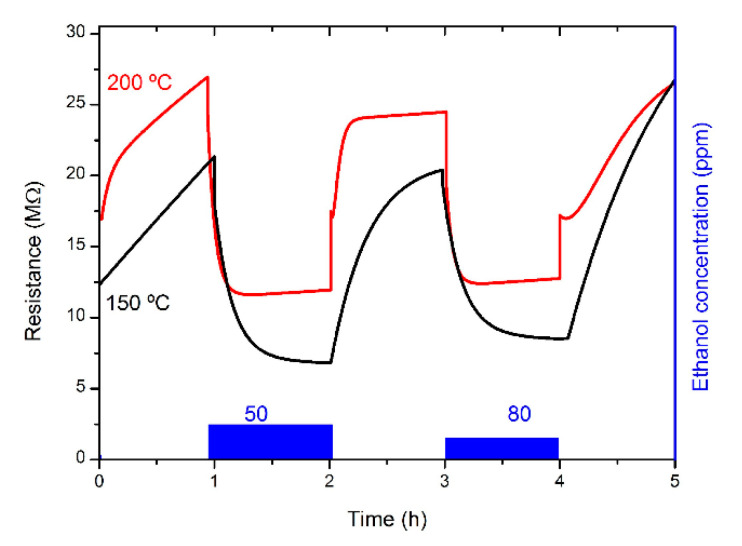
Resistance variation of a single NW-based sensor for ethanol in consecutive 1 h long pulses of different concentrations, measured at 150 °C.

First, we present the measurement of the sensors’ response towards ethanol gas diluted in dry SA. One-hour exposures to the gas of interest, followed by one-hour recovery in synthetic air, were used to study the processes of both adsorption and desorption of the gas by the sensing material. In Figure 11, the resistance variation is shown for a NW from Sample A, operating at 150 °C, with power consumption of just 2.3 mW. It is evident that the resistance of the sensors sharply drops when ethanol is present in the environment, in agreement with the reported reducing behavior of ethanol for n-type MOx sensors and with the proposed sensing mechanism, as reported for In_2_O_3_ elsewhere [4].

Moreover, the resistance variation shows a sizeable dependence on the ethanol concentration in the gas mixture, indicating the possibility of using this material for sensing purposes.

The relevant parameters of the sensors obtained from this kind of tests are summarized in Table 2. One can observe that the optimal operation temperature of this sensor is 200 °C. Although a minimum operational temperature of 150 °C is required for this sensor to provide a stable and consistent behavior, by increasing it to 200 °C the response time rises about one order of magnitude. On the other hand, the recovery is a much slower process, and not as sensitive to temperature variations within the measured range, indicating that the gas desorption is one of its main limiting factors, working under these conditions.

**Figure 12:**
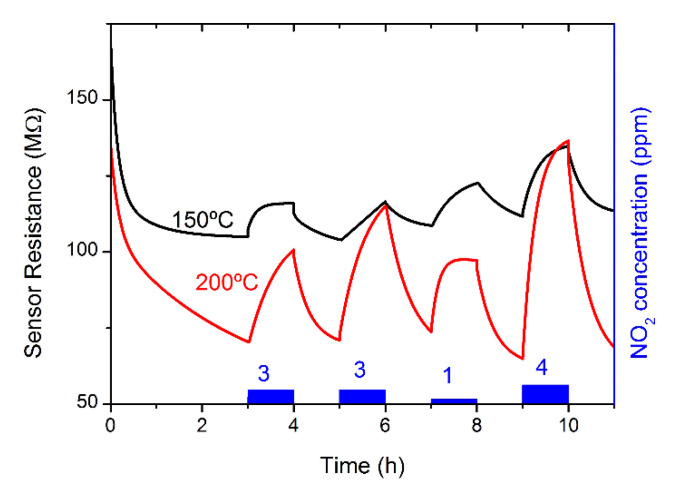
Sensor resistance of a single NW from Sample A for nitrogen dioxide consecutive pulses of different concentrations at two operation temperatures.

Similar tests performed in an atmosphere with NO_2_ diluted in SA reveal that this class of sensors is also efficient for oxidizing agents. As in the case of ethanol, the fabricated sensors exhibit a reversible response to different NO_2_ concentrations in a low temperature range (150–200 °C). Indeed, a good response was recorded in the range of 1 to 4 ppm of NO_2_, as shown in Figure 12. In addition, in this case the best results were attained at 200 °C, with a sizeable increase in the sensor response compared to an operation at 150 °C, and a power consumption that attained a value of only 3.2 mW.

In this investigated range of temperatures, attention can also be focused on the temporal evolution of the sensing process, which is rather insensitive to the thermal budget, lying in the range of several tens of minutes for both the onset of sensing and the recovery transient, as reported in Table 3.

It should be noted that working with such a slow response can cause interference in the measured response if the gas pulses are too close, as sometimes can be found in the experimental traces depicted in Figure 12.

It is also worth noting that parallel tests carried out in the same set-up and conditions on a single In_2_O_3_ NW-based gas sensor [4] resulted in a negligible response to NO_2_, always below 10% for the same range of concentrations. This occurrence is a valuable piece of information for the investigation of gas molecule trapping/desorption, which are the main physical mechanisms of the device’s function. We can suppose that both of these phenomena are driven by oxygen-vacancy diffusion to and from the NW surface, with typical rather long characteristic times, such as those we observed for an oxidizing specie such as NO_2_. Then, it is reasonable to deduce that the already mentioned Ga incorporation plays a fundamental role in triggering the efficiency of generation and/or diffusion of these vacancies, either by the tensile stress in the lattice, introduced by the replacement of In with Ga ions, or by an enhancement in vacancy generation required to reduce the aforementioned stress.

Even if the presented results are preliminary, using this multifold investigation we demonstrated the interesting potentiality of fabricating a novel nanomaterial for low-power gas sensing. Further advancements are foreseen, because work is underway to assess the stability of the sensors and to test additional potential gas targets. Long-term research will focus on attempting alternative routes for material fabrication, on exploring possible strategies to further reduce the response time of the devices, and on further reducing the power consumption by performing self-heating experiments while in the presence of gases. The evolution of this kind of research will retain the key requirement of low power consumption, as already attained in the fabricated prototypes, and the goal of devices that can operate at room temperature.

## 4. Conclusions

The fabrication of Ga-rich indium oxide nanowires and their use as an active material in gas sensing was demonstrated. The growth of (Ga_x_In_1-x_)_2_O_3_ NWs was carried out using the carbothermal metal-assisted VLS mechanism. This was achieved for different proportions of Ga_2_O_3_–In_2_O_3_ precursors to explore different Ga concentrations.

Structural characterization established that monocrystalline NWs of about 1 µm length and 100 nm diameter were grown in the <1 0 0> direction, irrespective of the precursor composition. Due to the different formation enthalpy of the precursors, the incorporation of Ga as a substitutional ion in the indium sites of a lattice close to that of In_2_O_3_ is very small, reaching a maximum of 5–6% of the metallic amount in the present conditions.

A set of gas sensor prototypes was fabricated by placing single NWs in contact with suspended membranes, with a buried heater. In this way, it was possible to operate the device at a temperature of 200 °C with a power consumption of 3.2 mW. Preliminary tests were carried out for both ethanol and nitrogen dioxide, demonstrating the good performance of this material as a low-temperature gas sensor. The demonstrated performance was better than that recorded for similar devices based on pure In_2_O_3_ single NWs.

## Figures and Tables

**Table 1:** Parameters evaluated from the XRD analysis: angle position of the (2 2 2) peak, lattice parameter and estimated composition of the NW assembly of the different synthesized samples.

SAMPLE	2*θ* (°)	*a* (nm)	*x* in (Ga_x_In_1-x_)_2_O_3_
Sample A	30.64	1.0109	0.025
Sample B	30.59	1.0118	<0.01
Sample C	30.67	1.0098	0.06
Sample D	30.71	1.0086	0.09

**Table 2:** Measured response and characteristic response and recovery times of a sensor based on a single (Ga_x_In_1-x_)_2_O_3_ NW from Sample A, operating at temperatures of 150 and 200 °C, for ethanol between 10 and 80 ppm. For comparison, parameters are included from similar tests with a comparable In_2_O_3_ NW sensor [4].

Operating Temperature (°C)	Δ*R*/*R* (%)	τ_res_ (s)	τ_rec_ (s)
150	33 [10 ppm]; 60 [80 ppm]	500	1150
200	35 [10 ppm]; 105 [80 ppm]	40	800
200 **(In_2_O_3_ NW)**	25 [10 ppm]; 40 [80 ppm]	160	2000

**Table 3:** Measured response and characteristic times of a single (Ga_x_In_1-x_)_2_O_3_ NW sensor operating at temperatures of 150 and 200 °C for nitrogen dioxide between 1 and 4 ppm.

Operating Temperature (°C)	Δ*R*/*R* (%)	τ_res_ (s)	τ_rec_ (s)
150	20	2260	2460
200	220	1980	2780
